# Compassionate Conservation and the Challenge of Sustainable Wildlife Management: A Survey of the Urban Public of China

**DOI:** 10.3390/ani11092521

**Published:** 2021-08-27

**Authors:** Zhen Miao, Qiang Wang, Xinyi Lu, Dongxiao Chen, Wei Zhang, Xuehong Zhou, Douglas Craig MacMillan

**Affiliations:** 1 College of Wildlife and Protected Area, Northeast Forestry University, Harbin 150040, China; miaozhen43566@163.com (Z.M.); luxinyi981125@163.com (X.L.); dongxiao_c@163.com (D.C.); 2 Key Laboratory of Wetland Ecology and Environment, Northeast Institute of Geography and Agroecology, Chinese Academy of Sciences, Changchun 130102, China; qwang@neigae.ac.cn; 3 Durrell Institute of Conservation and Ecology (DICE), University of Kent, Canterbury CT2 7NR, UK; dcm@kent.ac.uk

**Keywords:** sustainable wildlife management and conservation, compassionate conservation, animal welfare and rights, the public, China

## Abstract

**Simple Summary:**

Sustainable wildlife management (SWM), based on traditional practice supported with advances in scientific knowledge and evolving economic and social circumstances, has shaped the global approach to wildlife management and policy. In this paper, we report the findings of a large semi-structured questionnaire in China which investigated the attitude of the urban public toward sustainable wildlife management and wildlife conservation across a range of issues and identified the key socio-economic and demographic factor drivers for those attitudes. The survey was conducted from November 2018 to October 2020, across 15 cities randomly selected among China’s seven administrative geographic regions. The survey was initially conducted through face-to-face interviews, but later, due to COVID-19 restrictions, was completed via online questionnaires. The results show that the public are broadly supportive of the theory of SWM, but their enthusiasm is issue- and context-dependent. For example, on issues of “Animal Welfare and Rights,” “Wildlife Utilization and Captive Breeding,” and “Trophy Hunting”, the public demonstrate antagonistic views about SWM, demonstrating an affinity for “Compassionate Conservation”. We also found that demographic characteristics of the public can significantly influence attitude, with those respondents who are not vegetarian or religious, who have higher levels of education, or are younger in age being more likely to appreciate the rational science approach of SWM. Our research suggests that conservation organisations may need to adapt their management aims and practices to avoid direct conflict with the rising tide of animal rights sentiment. Furthermore, significant investment will be required to promote science-based conservation in social marketing on all social media platforms to help educate and engage the public with the science behind conservation management.

**Abstract:**

Sustainable wildlife management (SWM) is based on a synergy of traditional/local knowledge, advances in scientific knowledge, and fast-evolving economic and social circumstances. A widely accepted cornerstone of SWM globally is that conservation and utilization need to be effectively integrated, emphasizing the benefits humans can derive from biodiversity, thereby further encouraging people to protect and value wildlife though its management. However, with demand from biological resources growing at an unprecedented rate and the emergence of social media, conservationists must respond quickly to new challenges and conflicts associated with species management and public policy. For example, the rise of the “Compassionate Conservation” (CC) movement, fueled by social marketing and media, which promotes the welfare of individual animals, has introduced a set of challenges for conventional conservation management as it opposes most or all forms of wildlife utilization and hunting. CC advocates are increasingly influential at global and national policy levels; hence, it is imperative that conservationists are informed and aware of the future challenges from a rapidly changing global society. In this paper, we report the findings of a large semi-structured questionnaire in China which investigated the attitude of the urban public toward sustainable wildlife management (SWM) and wildlife conservation across a range of issues and identified the key socio-economic and demographic factor drivers for those attitudes. The survey was conducted from November 2018 to October 2020, across 15 cities randomly selected among China’s seven administrative geographic regions. The survey was initially conducted through face-to-face interviews, but later, due to COVID-19 restrictions, was completed via online questionnaires. A Likert seven-point scale method was used to score the public’s degree of agreement or disagreement for each question; a multivariate stepwise linear regression method was used to analyze whether the overall attitude of the respondents toward SWM and wildlife conservation was affected by their demographic characteristics; and a classification and regression tree (CART) was used to conduct an in-depth analysis of the issues with negative scores in the questionnaire, so as to understand how the respondents’ demographic characteristics affected the public’s attitude about such issues, which could supplement results obtained from the multivariate stepwise linear regression analysis. The results show that the public are broadly supportive of SWM, but only moderately so. On issues of “Animal Welfare and Rights,” “Wildlife Utilization and Captive Breeding,” and “Trophy Hunting”, the core concerns of the “Compassionate Conservation” movement and the overall public view are more antagonistic to conventional SWM. We also find specific demographic characteristics significantly influence attitudes about SWM, with vegetarians, those with religious beliefs, and with lower educational standards demonstrating weaker support for SWM. For younger people, “Animal Welfare and Rights” is a special concern, hence, we identify this as a key issue to be addressed for SWM and conservation in the future. Our research suggests that conservation organisations may need to adapt their management aims and practices to avoid direct conflict with the rising tide of animal rights sentiment, especially among the young. Furthermore, significant investment will be required to promote science-based conservation in social marketing on all social media platforms to help educate and engage the public with the science behind conservation management.

## 1. Introduction

Sustainable wildlife management (SWM) refers to the sound management of wildlife species to sustain their populations and habitat over time based on an effective synergy of scientific and traditional/local knowledge, considering the socioeconomic needs of human populations [1]. The widely accepted cornerstone of SWM globally is that conservation and utilization need to be effectively integrated, emphasizing the benefits humans can derive from biodiversity, thereby further encouraging people to protect and value wildlife through its management [2]. In recent years, with the continuous development of society, SWM is moderated and influenced by evolving economic and social trends and expectations. However, with the emergence of social media and the globalization of conservation management conflicts, conservation managers and domestic policy makers must respond more quickly to new challenges and conflicts that arise from the concerns of the global community [3].

“Compassionate Conservation” (CC) is a rapidly emerging movement in the field of conservation science which promotes and advocates equal protection of all animal individuals based on “sympathy and compassion” under the principles of “do no harm; individuals matter; inclusivity of individual animals; and peaceful coexistence between humans and animals” [4]. These principles of CC and the underlying values attached to them represent a significant and growing challenge to traditional conservation aims, policies, and practices that are focused scientific approaches to conserving species and habitats [5]. The contrasting conservation stance between traditional conservation and CC raises profound questions for the role of science and, for example, the acceptability of hunting as foundations of sustainable wildlife conservation and management [6]. When “Cecil the Lion” was killed by a trophy hunter in the Hwange National Park in Zimbabwe in 2015, there was a rapid and coordinated online campaign to end trophy hunting globally [7]. Even in countries such as South Africa, which has hitherto been a strong proponent of sustainable wildlife management and use, has proposed measures in 2021 to prohibit the captive breeding of all African lions for wildlife tourism, trophy hunting, and traditional medicine [8]. Lethal control of invasive species [9] and the legal wildlife trade [4] are also rapidly attracting organised and vehement opposition, even when there are strong scientific underpinnings for such actions [10]. It is therefore imperative that wildlife managers become more informed about the current and impending challenges posed by the attitudes and values of a fast-evolving global society toward wildlife.

In China, “Compassionate Conservation” has rapidly attracted support with the wide-spread promotion of various animal causes and extensive publicity about animal rights and cruelty [3]. For example, in March 2012, several airlines including China Southern Airlines, Air China, and China Eastern Airlines announced the suspension of transporting animals for experimental research, as the public began to overwhelmingly oppose the use of animals for experiments under the widespread publicity of PETA [9]. Vegetarianism has also been growing strongly in China, supported and enabled through social media and conventional advertising campaigns urging people to give up meat on the basis that animals have equal rights to people [11]. Scientific knowledge appears to hold little sway with actions and policies promoted by CC, often heedless of negative repercussions for the animals themselves, other animals and species, and for ecological processes. For example, in April 2016, animal rights campaigners purchased 380 artificially bred blue foxes (*Alopex lagopus*) and raccoon dogs (*Nyctereutes procyonoides*) at a farm in Hebei Province, and released these animals randomly in the Beijing area [12]. However, the animals did not have the ability to survive “in nature”, and resorted to killing livestock, household pets, and poultry, and causing considerable distress to local people.

The rise of the CC movement, fueled by social marketing and media, appears likely to pose major challenges to SWM orthodoxy, focused as it has been on biodiversity, species conservation, and ecological balance and often reliant on some form of direct or indirect utilization [5,13]. Wildlife conservation and management is therefore set to become an even more complex issue in China and around the world. In particular, issues of hunting, especially trophy hunting [14], and wildlife utilization for medicines or food [15,16], which often can provide key economic incentives for local people to actively participate in species and habitat conservation and are the mainstay for conservation management practices and funding at local levels [17,18], are likely to come under pressure, threatening the integrated approach to conservation and sustainable rural development. In addition, the use of wildlife for research will also be threatened, even if this research can improve scientific information needed for more effective wildlife conservation [19].

At this point in time, erosion of public support for SWM has therefore become an important and urgent conservation issue, but there is a large gap in our knowledge about public attitudes towards SWM and how this may be influenced by CC in China and elsewhere. In this paper, we report the findings of a large semi-structured questionnaire in China where we systematically investigated the attitudes of the urban public toward sustainable wildlife management and wildlife conservation across a range of issues, particularly including animal welfare and rights, wildlife utilization, and trophy hunting, which are fundamental conflict issues between CC and SWM. We also analyse the role key socio-economic and demographic factors have in terms of influencing those attitudes with a view to achieving a better understanding of the key challenges to SWM and conservation management more generally and to propose more scientific solutions. It is worth noting that for CC in this study, we not only want to study the CC as a specific conservation science movement, but rather to focus more on the broader public impact of its underlying principles.

## 2. Materials and Methods

### 2.1. Subject, Location of Data Collection

**Figure 1:**
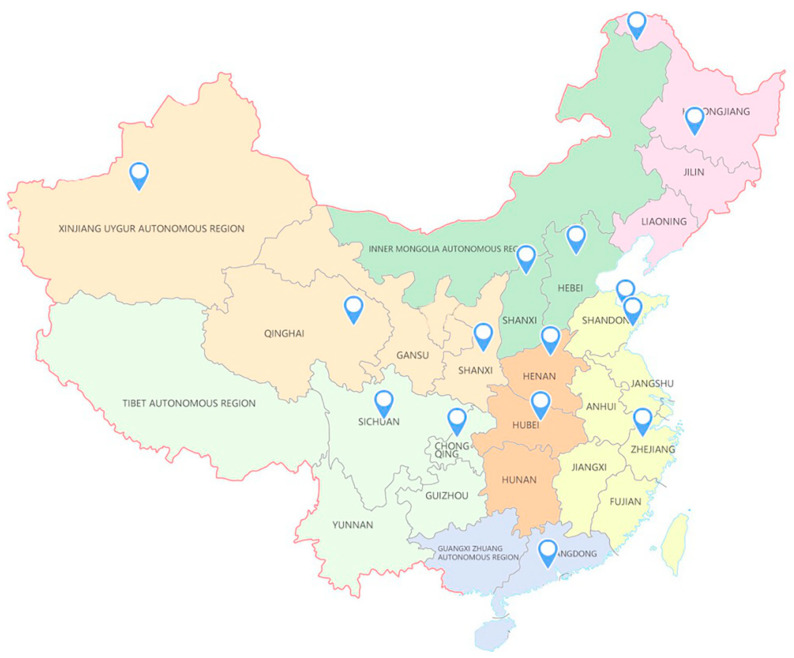
Survey area for attitudes about sustainable wildlife management and conservation (Northwest China is marked in beige; Southwest China is marked in light green; North China is marked in dark green; Central China is marked in orange; South China is marked in purple; East China is marked in yellow; Northeast China is marked in pink).

From November 2018 to October 2020, the urban public in 15 cities in China were chosen as the research subjects (hereinafter referred to as the public), to conduct research on attitudes toward the theory of SWM and wildlife conservation. Provinces in China are divided into seven administrative geographic regions, namely North China, East China, Central China, South China, Southwest China, Northwest China, and Northeast China, according to multidimensional factors such as geographic location, history, national culture, etc [20]. We used systematic sampling in 15 major Chinese cities covering all seven regions. These were Beijing and Datong in North China; Hangzhou, Yantai, and Zhucheng in East China; Wuhan and Zhengzhou in Central China; Guangzhou in South China; Chongqing and Chengdu in Southwest China; Xining, Xi’an, and Xinjiang Uygur Autonomous Region He Jing County in Northwest China; Harbin and Genhe in Northeast China (Figure 1).

Initially, random field surveys to interview respondents face-to-face were carried out, but later surveys took the form of online questionnaires distributed through the “Questionnaire Star” platform due to the outbreak of the Corona Virus Disease (COVID-19). In this study, 1921 questionnaires were collected in 11 cities (Beijing, Datong, Hangzhou, Yantai, Zhucheng, Guanzhou, Wuhan, Harbin, Genhe, Xining, Xinjiang Uygur Autonomous Region He Jing County, Chongqing) through on-site interviews. Excluding incomplete questionnaires, there were 1576 valid questionnaires, with a completion rate of 82.0%. A total of 932 questionnaires were collected online in four cities (Guangzhou, Zhengzhou, Xi’an, Chengdu), of which 734 were valid questionnaires, with a completion rate of 78.8% (the sample breakdown across the fifteen cities is shown in Table S1, Supplementary Materials). All surveys were voluntary and anonymous, and the respondent’s informed consent was obtained before the survey started.

### 2.2. Questionnaire

A semi-structured questionnaire was designed to evaluate public attitudes toward the theory of SWM and wildlife conservation across a range of issues. The questionnaire consisted of 27 questions, divided into two parts. In the first part, we used seven questions to ascertain the demographic characteristics of the respondents, including gender, city of residence, age, education, monthly salary, and recorded whether the respondent was a vegetarian (vegetarianism mentioned in this study refers to refusal to eat one or more animal products), or had religious beliefs. In the second part, an attitudes scale regarding SWM, was introduced [3]. The questions were connected to various contemporary social issues related to the wildlife conservation and management in China today such as Release [21], Animal Welfare and Rights [22], Vegetarianism [23], and Trophy Hunting [24]. To assess respondent attitudes quantitatively we incorporated twenty questions in total, which for the analysis we grouped into seven categories (Animal Release, Animal Welfare and Rights, Wildlife Utilization and Captive Breeding, People and Wildlife Management, Vegetarianism and Wildlife Conservation, the role of Public Attitudes to Wildlife Conservation, and Trophy Hunting).

A Likert seven-point scale was used to assess respondents’ degree of agreement or disagreement with SWM for each question from both forward (questions 4, 5, 6, 8, 9, 12, 13, 16, 19, 20) and reverse (questions 1, 2, 3, 7, 10, 11, 14, 15, 17, 18) angles (Table 1). For questions in the forward angle, the score ranged from −3 (strongly disagree) to +3 (strongly agree); for reverse questions, they were scored in the opposite fashion [25]. This was done to encourage respondents to consider their replies for each question. After the survey, the average value of all questions and the seven categories of issues were calculated to generate a comprehensive score. The average score obtained represented the respondent’s attitude toward SWM and wildlife conservation. The higher the final score, the more consistent in broad terms the respondent’s attitude and cognition were in line with the theory of SWM. For example, a strongly negative average score for a question would suggest a potential conflict with SWM theory or practice.

### 2.3. Statistical Analysis

All data obtained from the questionnaire was processed and analyzed using SPSS 26.0 (IBM Corporation, Armonk, NY, USA). Descriptive statistical methods were used to analyze the demographic characteristics of the respondents, such as gender, city of residence, age, education, monthly salary, vegetarianism, and religious beliefs. In addition, considering that the degree of city development may have an impact on the attitude of respondents, we classified the 15 cities where the respondents are located according to the degree of city development, and descriptive statistics were performed. According to the “2020 China City Business Charm Ranking List” released by China Business News, Chinese cities can be divided into first-tier cities, new first-tier cities, second-tier cities, third-tier cities, and below based on the commercial resource concentration, urban hub, urban people’s activity, diversity of lifestyle, and future plasticity [26]. The average value for the 20 questions and seven categories of issues were also calculated to obtain the average total score of respondents’ attitudes toward SWM and wildlife conservation for subsequent analysis. Multivariate stepwise linear regression was used to analyze the impact of demographic variables on the overall attitudes toward SWM and wildlife conservation and reveal the importance of each variable in explaining differences in attitudes using the average total score of respondents’ attitudes toward SWM; and wildlife conservation was the dependent variable, with demographic characteristics such as gender, age, and education as the independent variables, and dummy variables set [27]. In this study, *p* < 0.05 was considered significant. In addition, we conducted an in-depth analysis of the seven categories of issues with negative scores using CART analysis and post-pruned them to establish whether the respondents’ demographic characteristics affected the public attitudes about these issues. CART was used to supplement the results obtained from the multivariate stepwise linear regression, by categorical branch analysis of the data [28]. Post-pruning solves the problem of decision tree over-fitting and refers to pruning the branches of a fully grown decision tree through evaluation criteria to generate an optimal decision tree model [29].

## 3. Results

### 3.1. Demographics

The proportion of men and women was balanced, 52.2% and 47.8% respectively, which is consistent with the overall ratio of men and women in China [30]. The proportion of respondents aged 21–30 years old (25.5%), 31–40 years old (25.4%), and 41–50 years old (24.7%) is higher than the population age structure of the Chinese society, while the proportion of respondents younger than 20 years old (16.5%), 51–60 (6%) and older than 61 years old (1.8%) is lower than the population age structure of Chinese society [30]. In this study, the educational background of the respondents was higher than the average level in China [30]. Respondents who had high school (secondary school), college, and undergraduate degrees accounted for a larger number with a relatively even distribution, 29.1%, 30.8%, and 26.9%, respectively; those with junior high school and below and postgraduate degrees took up smaller proportions, 8.2% and 4.9%, respectively. Most of the respondents (41.6%) had monthly salaries below 4000 yuan. Most of the respondents were non-vegetarians (82.8%), and most had no religious belief (85.8%). Nearly half (59.7%) came from first-tier cities and new first-tier cities (full details in Table S2, Supplementary Materials).

### 3.2. Overall Attitude and Influencing Factors of Public toward SWM and Wildlife Conservation

**Figure 2:**
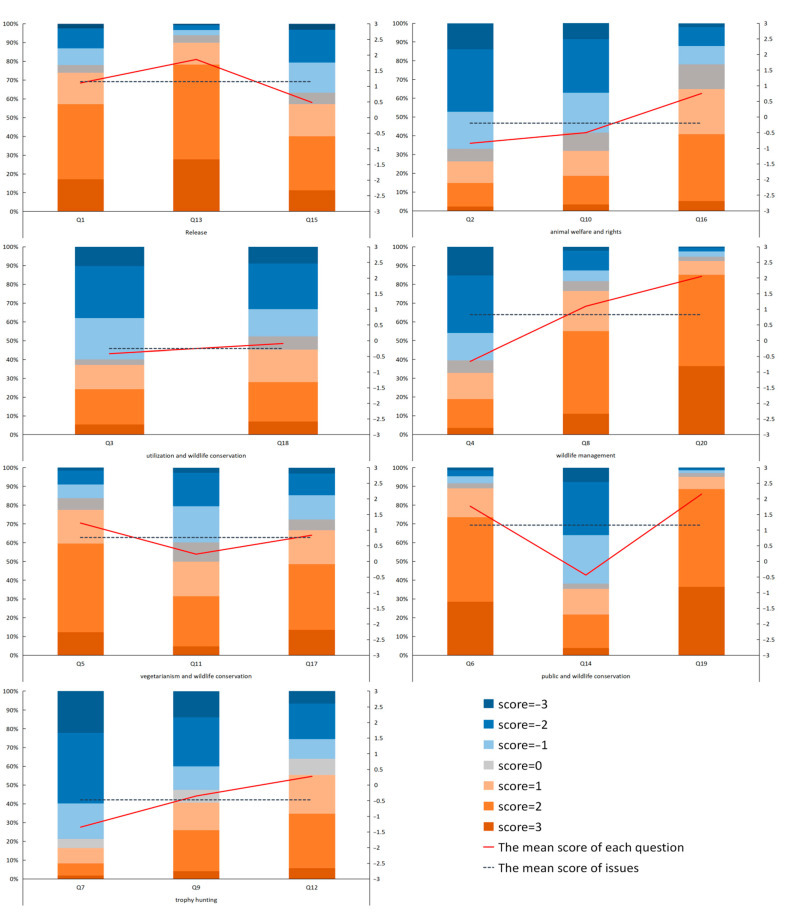
Attitudes of the public toward seven categories of issues and twenty questions in the questionnaire (the histogram represents the frequency distribution of respondents’ scores on the 20 questions; the red broken line represents the mean score of the respondents on each question; the black dotted line represents the mean score of the respondents for each category of issue. If the score is greater than 0, this means that the public’s perception on this issue/question is consistent with SWM; if the score is less than 0, this means that the public’s perception on this issue/question is contrary to accepted SWM theory and/or practice).

The average score for public attitudes toward the individual questions and the combined seven categories was estimated from the Likert scale (Figure 2). The higher the score, the more the respondents support SWM. The overall average score on the range −3 to 3 was 0.458, indicating that on average the public were mildly supportive of SWM. In terms of the seven categories of SWM and wildlife conservation issues, the average score was positive for “Animal Release” (1.147), “People and Wildlife Management” (0.828), “Vegetarianism, and Wildlife Conservation” (0.763), “Public Attitude to Wildlife Conservation” (1.157), while the scores of “Animal Welfare and Rights” (−0.199), “Wildlife Utilization and Captive Breeding” (−0.255), and “Trophy Hunting” (−0.475) were negative.

Looking at responses to individual questions, public attitudes for most questions were positive. For “Animal Release”, the scores of Q1 (1.105), Q13 (1.856), Q15 (0.481) were both higher than 0, with the score for Q13 higher than the average score of the “Animal Release”. For “Animal Welfare and Rights”, the scores of Q16 (0.750) were positive, while the scores of Q2 (−0.844) and Q10 (−0.504) were negative, with the score for Q16 higher than the average score of this issue. For “Wildlife Utilization and Captive Breeding”, the scores of Q3 (−0.415) and Q18 (−0.095) were all lower than 0, with the Q18 higher than the average score of “Wildlife Utilization and Captive Breeding”. For “People and Wildlife Management”, the scores of Q8 and Q20 were higher than 0, while the score of Q4 was lower than 0 and the average score of “People and Wildlife Management”. For “Vegetarianism and Wildlife Conservation”, the scores of Q5 (1.225), Q11 (0.230), and Q17 (0.836) were higher than 0, with Q11 lower than the average score of “Vegetarianism and Wildlife Conservation”. For “Public Attitude to Wildlife Conservation”, the scores of Q6 (1.763) and Q19 (2.148) were higher than 0, while Q14 (−0.440) was lower than 0 and the average score of “Public Attitude to Wildlife Conservation”. For “Trophy Hunting”, the score of Q12 (0.276) was higher than 0, while the scores of Q7 (−1.348) and Q9 (−0.352) were lower than 0, with Q7 lower than the average score of “Hunting Issues” (the average and extreme scores for each question are shown in the Supplementary Materials, Table S3).

A multivariate stepwise linear regression analysis was conducted in order to study the influencing factors of the public’s overall attitudes toward SWM and wildlife conservation (Table 2). The average total score of respondents’ attitudes toward SWM and wildlife conservation was the dependent variable, and demographic characteristics (education, age, gender, vegetarianism, religious belief, monthly salary, level of city) as independent variables. The results show that vegetarianism, education level, religious beliefs, age, gender, and city size/level all had a significant impact on the public attitudes toward SWM and wildlife conservation.

The overall average score of the public’s attitudes toward SWM and wildlife conservation was significantly positively correlated with education (*p* = 0.000 < 0.01), and significantly negatively correlated with level of city (*p* = 0.000 < 0.01), age (*p* = 0.000 < 0.01), gender (*p* = 0.000 < 0.05), religious beliefs (*p* = 0.000 < 0.01), and vegetarianism (*p* = 0.000 < 0.01). The higher the education level of the public, the higher the support for SWM attitudes for wildlife conservation; members of the public who had relatively low average total score generally came from lower-level cities, were older, were female, had religious beliefs, and were vegetarian.

### 3.3. Attitude and Influencing Factors of Public toward Issues with Negative Scores

We investigated in more detail the three issues where public attitudes toward SWM and wildlife conservation were negative: “Animal Welfare and Rights”, “Wildlife Utilization and Captive Breeding”, and “Trophy Hunting”, as these issues are the fundamental conflict issues of the CC movement and are perhaps of most immediate concern to current wildlife conservation practices and policies in China. An in-depth analysis of individual responses was therefore conducted using CART.

#### 3.3.1. Animal Welfare and Rights

The frequency statistics of the three questions directly relating to Animal Welfare and Rights (Q2, Q10, and Q16) are shown in Figure 2. According to the results, in Q2, the number of people with a negative attitude (score < 0) accounted for 66.9%, the number of people with a neutral or indifferent attitude (score = 0) accounted for 6.8%, and the number of people with a positive attitude (score > 0) accounted for 26.3%. In Q10, the number of people with a negative attitude (score < 0) accounted for 58.4%, the number of people with a neutral or indifferent attitude (score = 0) accounted for 9.8%, and the number of people with a positive attitude (score > 0) accounted for 31.9%. In Q16, 21.8% had a negative attitude (score < 0), 13.2% had a neutral or indifferent attitude (score = 0), and 64.9% had a positive attitude (score > 0).

**Figure 3:**
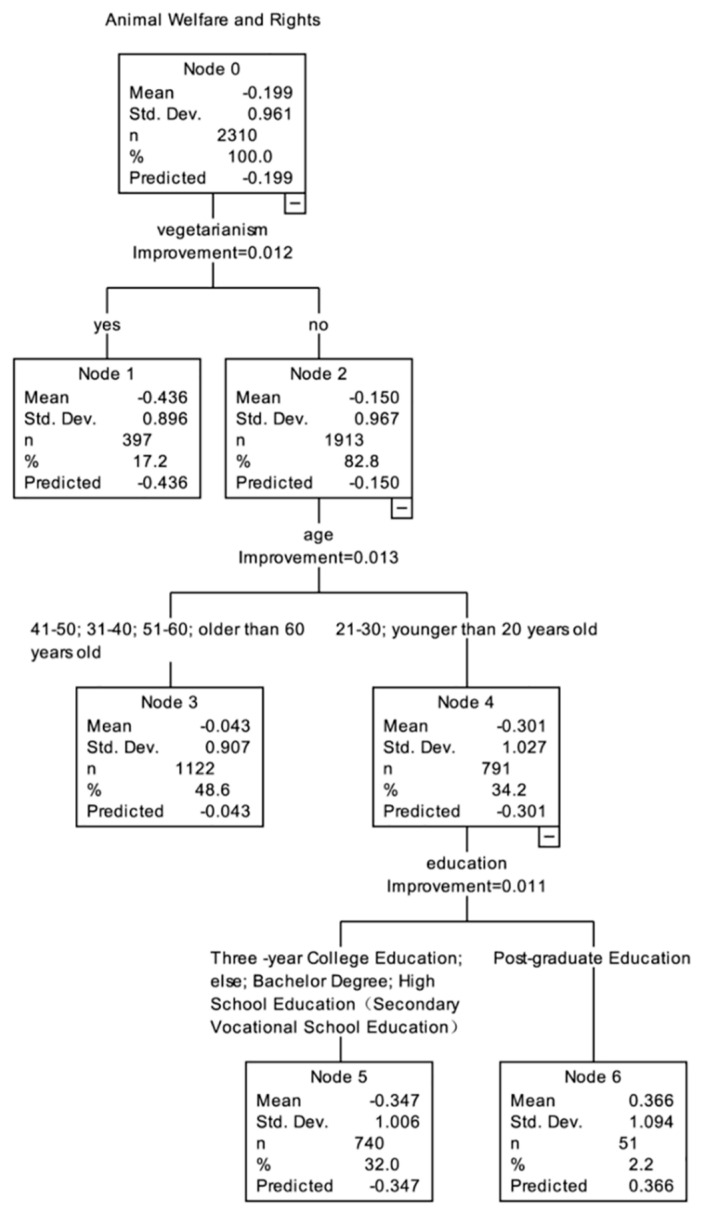
The classification and regression tree model for the issue of Animal Welfare and Rights.

The method of CART followed by post-pruning (Figure 3) was used to analyze the influencing factors of the issue of “Animal Welfare and Rights”. Generally speaking, vegetarianism, age, and education explained the difference best regarding public attitudes about “Animal Welfare and Rights”. Overall, non-vegetarians under 30 years of age with a postgraduate degree had the most consistent support for SWM on the issue of “Animal Welfare and Rights.” The detailed results showed that “vegetarianism” was the first branch point in the decision tree, and therefore the most important influencing factor. The score of vegetarians (−0.436) was lower than that of non-vegetarians (−0.150). For the non-vegetarian public, “age” was the second branching factor. The score of the public under 30 (−0.301) was lower than that of the public over 30 (−0.043). Among the public under the age of 30, “education” was the influencing factor of the third branch point. The score of the public who had received postgraduate education (0.366) was higher than that of the public with other degrees (−0.347).

#### 3.3.2. Wildlife Utilization and Captive Breeding

The frequency statistics of Q3 and Q18 were counted to further analyze the specific distribution of the respondents’ scores on the issue of “Wildlife Utilization and Captive Breeding” (Figure 2). The results showed that in Q3, the number of people with a negative attitude (score < 0) accounted for 59.9%, the number of people with a neutral or indifferent attitude (score = 0) accounted for 2.9%, and the number of people with a positive attitude (score > 0) accounted for 37.2%. In Q18, 47.7% had a negative attitude (score < 0), 7.1% had a neutral or indifferent attitude (score = 0), and 45.2% had a positive attitude (score > 0).

**Figure 4:**
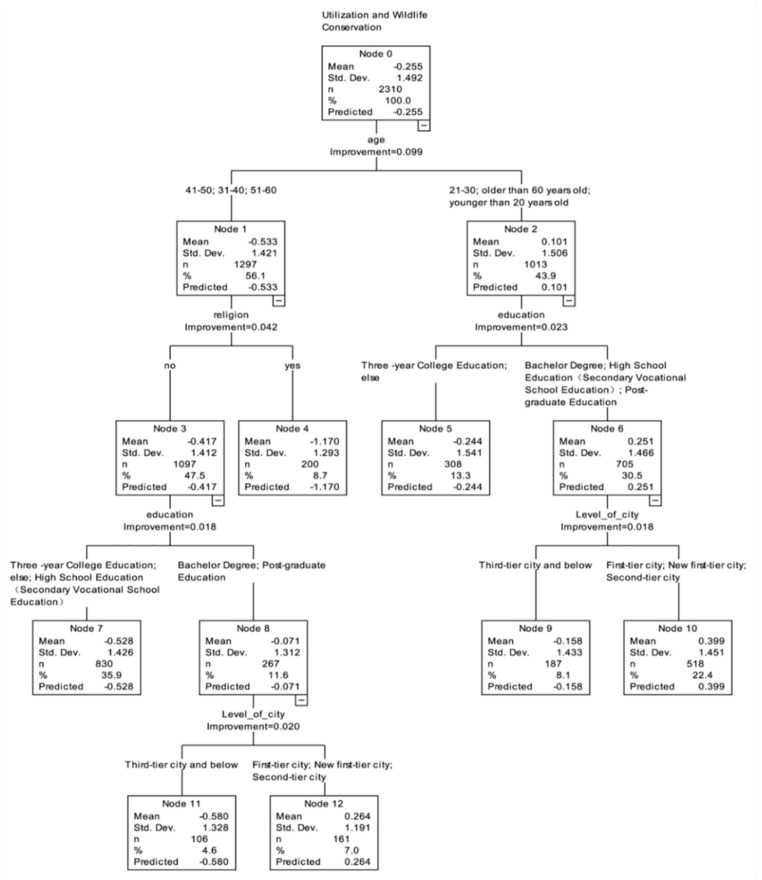
The classification and regression tree model for the issue of Wildlife Utilization and Captive Breeding.

The CART analysis identified that “age” was the first branch point in the decision tree, and therefore the most important influencing factor (Figure 4), with members of the public under 30 or over 60 (0.101) more supportive of SWM compared to the public aged between 31–60 years old (−0.533). For the former group, “education” was the influencing factor at the second branch point, with those respondents with a college degree or below junior high school degree (−0.244) with lower support for SWM than respondents with other degrees (0.251). For the latter public group, “level of city” was the influencing factor at the third branch point, with the average score of the public from third-tier and lower cities (−0.158) lower than that of the public in other cities (0.399). For the public aged between 31–60 years old, “religious belief” was the influencing factor of the second branch point. The score of the public with religious beliefs (−1.170) was lower than that of the public without religious beliefs (−0.417). For the public with no religious belief, “education” was the third factor influencing the third branch, with the score of the public with undergraduate and postgraduate degrees (−0.071) higher than that of the public with other degrees (−0.528). For the public with undergraduate and postgraduate degrees, “level of city” was the influencing factor of the fourth branch point, and the scores of the public from third-tier cities and below (−0.580) were lower than those of other cities (0.264).

#### 3.3.3. Trophy Hunting

The frequency statistics of the three questions directly related to Trophy Hunting (Q7, Q9, and Q12) are shown in Figure 2. The results show that in Q7, the number of people with a negative attitude (score < 0) accounted for 78.8%, the number of people with a neutral or indifferent attitude (score = 0) accounted for 4.9%, and the number of people with a positive attitude (score > 0) accounted for 16.4%. In Q9, 52.4% had a negative attitude (score < 0), 6.9% had a neutral or indifferent attitude (score = 0), and 40.6% had a positive attitude (score > 0). In Q12, 35.9% had a negative attitude (score < 0), 8.7% had a neutral or indifferent attitude (score = 0), and 55.4% had a positive attitude (score > 0).

**Figure 5:**
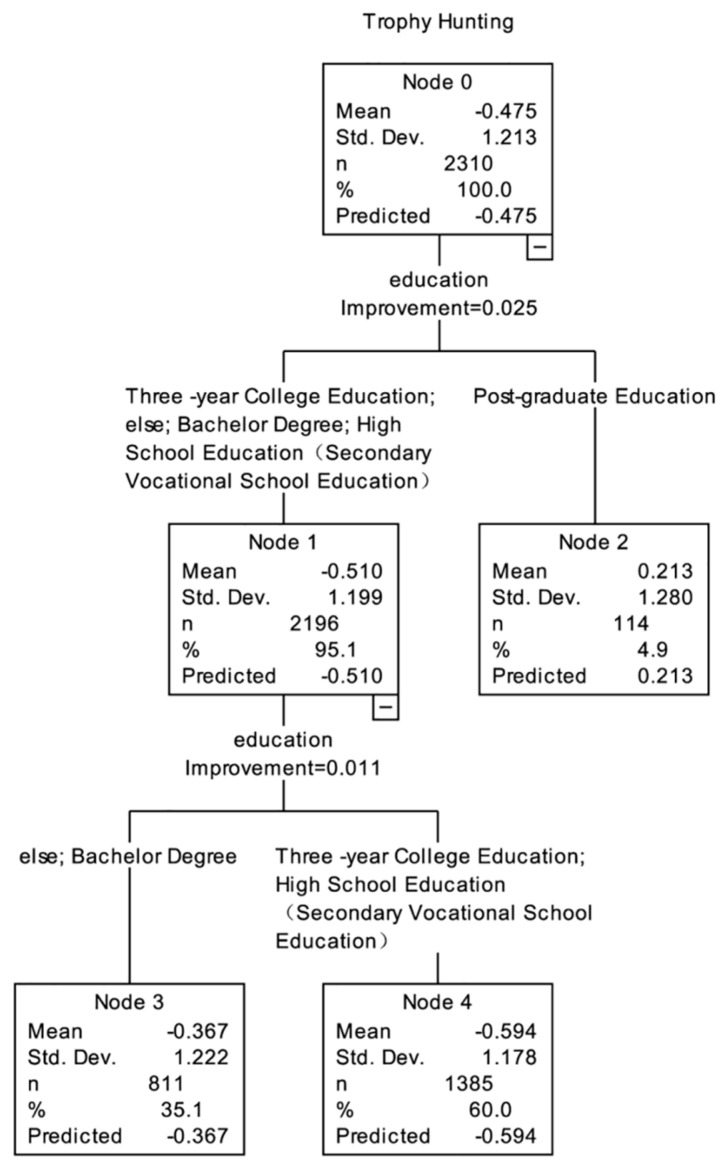
The classification and regression tree model for the issue of Trophy Hunting.

Results from the CART analysis showed that “education” was the first branch point in the decision tree, and therefore the most important influencing factor (Figure 5). The score of the public with a postgraduate degree (0.213) was higher than that of the public with other degrees (−0.510). For the public with a junior high school degree or below, a high school (secondary school) degree, a college degree, and a bachelor degree, “education” was the influencing factor of the second branch point, and those with a bachelor degree scored (−0.367) higher than the public with other degrees (−0.594). In general, the public with a postgraduate degree were more in agreement with SWM on the issue of “Trophy Hunting”.

## 4. Discussion

Our survey demonstrates that overall support for SWM practices and principles across a range of seven distinctive wildlife management and conservation issues is positive among the general public in China. This result is broadly similar to an earlier investigation of attitudes among Chinese college students [3]. Support for SWM was markedly different across different issues, with most support for a scientific-led approach to species management, greater recognition of the impact on people, and the promotion of ecological integrity and the protection of species. For example, the public are most strongly opposed to unscientific and random animal release. It is interesting to note that within each issue, there was evidence from the individual questions that public support for SWM practices and policies is nuanced and conditional, depending on the motive for utilization, or the impact of the activity on animal welfare. Support for SWM is also strongly determined by demographic characteristics, especially education level and age, although the relationship with demographics differs depending on the issue concerned. A key finding is the striking contrast in the lack of support for SWM across three issues most closely associated with “Compassionate Conservation”. Our discussion therefore focuses on these three issues and how conservation management and policy can adapt or mitigate the conflicts that might arise in China and elsewhere.

### 4.1. Sustainable Wildlife Management and Compassionate Conservation

#### 4.1.1. Animal Rights and Welfare

The results of this study strongly indicate that the urban public are supportive of animal rights. This places Chinese opinion, at least among the urban population, firmly in alignment with the global trend toward stronger support for animal rights [4]. For example, in Q2, the statement that animals should have equal rights with humans was supported by 67% of respondents. Previous studies revealed similar findings, that is, the public are becoming increasingly concerned about animal rights with the rapid development of Chinese society and the continuous influence of the CC movement [9].

Similar to animal rights, the urban public has also shown equivalent support for animal welfare. In Q16, 65% of respondents supported animal welfare should be improved in captive breeding operations. The equally strong support of respondents for both animal welfare and animal rights may be derived from some ambiguity amongst the public arising from media publicity [31]. Earlier studies have revealed this possibility. For example, Dolby (2018) showed that due to one-sided publicity by some animal organizations, many people of the American public mistakenly regard animal rights as a means to promote animal welfare and are unaware of the ultimate and deeper consequences of advocating for animal rights [32]. Animal welfare refers to animals under human control being given adequate care to ensure the safe and humane use of animals [33], which is an essential aspect of sound sustainable wildlife management [34]. In contrast, animal rights refer to all animals having their ethical status, believing that the elimination of all forms of animal utilization is the best way to protect animals, which has the same philosophical basis as animal liberation [35]. Consideration of demographic factors also suggests that there is considerable complexity with the overall results, with younger people and vegetarians most clearly supportive of animal rights with respect to human rights [36,37]. Respondents with higher educational attainment were, however, less supportive [38,39].

#### 4.1.2. Wildlife Utilization and Captive Breeding

Wildlife utilization has a long tradition in China, but the use of wildlife—even when it is legal and sustainable—has become controversial in recent years [40]. Our results suggest significant opposition in China to wildlife utilization (including direct utilization and indirect utilization), with 59.9% of respondents of the view that the wildlife conservation and utilization cannot be mutually beneficial; believing that wildlife utilization is antagonistic to their view of conservation. In this respect, China is also following a trend in most of the advanced western countries. For example, research by Tisdell (2007) et al. [41] shows that most of the Australian public have a relatively strict attitude about wildlife utilization and do not accept that sustainable utilization can promote wildlife conservation. The use of commercially captive breeding of wildlife and its products alone did not significantly increase public support for wildlife utilization. For example, in Q18, 47.7% of respondents believe that all wild animals and their products including captive breeding should be prohibited. Although there are studies have demonstrated that commercially captive breeding industry can guarantee good animal welfare, and in specific conditions, it can also become one of the effective methods to protect wild population while meeting human needs [42,43], our results show that the Chinese public still have an inherent negative impression about the captive breeding of wildlife.

Our results indicate that attitudes about Wildlife Utilization and Captive Breeding, compared to other issues, were more complex and nuanced, with support for SWM influenced by four independent demographic variables in the CART analysis. “Age” was the most significant influencing factor, with older and younger respondents more supportive of utilization. Respondents with most negative views about utilization tended to be less educated and have strong religious beliefs [44].

The relationship between age and attitudes toward SWM indicates that age cohorts have distinctive attitudes. Kendall (2006) et al. [36] indicates that the influence of age on public attitude about animals may have a cohort effect, in which people who have experienced a common history often share common beliefs and attitudes. In China, where older people in our survey grew up in a time where wildlife conservation was achieved through strict protection, SWM based on the principle of effective integration of sustainable utilization and conservation may be somewhat contradictory to them, and this may explain why this cohort were less supportive of SWM. Although young people are the main user groups of social platforms and hence more exposed to animals rights campaigning, they also have more advanced scientific education through schooling and, perhaps, are more able to grasp the need to balance compassion with scientific management [45].

#### 4.1.3. Trophy Hunting

Trophy hunting is one of the most controversial and high-profile issues in contemporary global conservation, with opposition amplified by social media coverage that has focused on the fate of individual animals [24,26]. Although trophy hunting was effectively banned in China in 2006, there is an active debate around the need to control populations of large wild animals causing economic damage, with trophy hunting seen as also a way of promoting local livelihoods through hunting [24].

In our study, 79% of the public support the view that “trophy hunting is cruel and inhumane”, but over 55% also supported well-managed trophy hunting. These apparent contradictions may be due to the specific aspects of trophy hunting, that have been highlighted in social media such as the motivation of the participant (only for trophy, not food or population management). For example, the lion “Cecil” was especially “vile” because the hunter was only interested in the trophy [24]. However, there are many examples of well-managed trophy hunting linking conservation to the economic interests of local people, providing key incentives for local people to actively protect species and the habitat, greatly contributing to the conservation and management of wildlife populations [14,18]. Byrd (2017), for example, found the American public were willing to accept hunting if the motivation was food and/or population management, whereas only a small minority accepted hunting for trophy purposes [46]. Therefore, if hunting is to remain an integral component of SWM, then authorities must invest in education and marketing strategies and campaigns that emphasise the strong scientific necessity of trophy hunting and the conservation benefits and economic benefits it provides. This is particularly important in schools where the role of trophy hunting in maintaining ecological balance and sustainable management would help children develop their capacity to consider trophy hunting holistically as opposed to the unfiltered messaging they are exposed to on social media [47].

### 4.2. Other Considerations for Conservation Management and Policy

Our survey focused exclusively on the attitudes of people living in large cities, the most numerous and affluent segment of Chinese society (China’s urban population accounted for 60.6% in 2019 [30]). Urban dwellers are, however, also the most removed from key issues wildlife managers and rural communities are facing such as wildlife conflicts (predation, crop damage, loss of life, etc.) and the need to alleviate poverty by providing income from wildlife management activities [48]. In some ways, the growing chasm between those contrasting communities (rural and urban) and their views about wildlife is perhaps the greatest challenge facing conservation [49]. Some countries, with a more rural-based population, have found it easier to combine conservation with sustainable use. For example, in 1996, Namibia approved the establishment of a community-based natural resource management (CBNRM) system. In this system, local residents directly benefit from wildlife management and utilization by having the right to use wildlife and tourism. Positive economic incentives have promoted wildlife conservation in Namibia. Since the implementation of the CBNRM system, the amount of wildlife in Namibia has increased dramatically. For example, the number of black rhinos has more than tripled, and the number of elephants has also increased from 7500 in 1995 to more than 20,000 today [7]. While this demonstrates that conservation and utilization can work, it may not be a sustainable way forward in more urban-centric societies where ideas and attitudes are being shaped and influenced by feelings and values rather than more utilitarian concerns. Education must therefore play a key role, providing balanced information on SWM to help counter the stark, often shocking imagery of animal rights groups on social media [50]. In particular, the education systems can provide students the opportunity to understand the complex nature of sustainability and the challenges facing managers with regards to animal conservation.

A large number of studies suggested that scientific education and information helped to improve individuals’ attitudes about wildlife conservation and management and strengthened people’s ability to discern misleading information and understand scientific conservation information [50]. Education was an important factor that affects public attitudes in our survey, with a general finding that educational attainment is positively correlated with support for SWM. Similar results have been found elsewhere [28,29], with education likely to increase appreciation and understanding of scientific rationales for management over feelings or belief-based systems [37]. However, the relationship between education and SWM is nuanced and it is unlikely to be the case that rational arguments and a science education will continue to play a hegemonic role on attitude formation among future generations that are growing up with a more reactive, emotional world where “world views” are increasingly shaped by superficial education by social media [51]. For example, by exploring and understanding the negative aspects of “kindness or love of animals” to other species, such as the introduction of cute non-native species [14] and the release of American mink from fur farms [*Neovison vison*], of native fauna [52].

More targeted education for groups with different demographic characteristics is needed according to the results of this study [53]. For example, more comprehensive and easy-to-understand environmental education materials can be designed for groups with a low level of education so as to help them understand the connotation of the theory of SWM quickly and comprehensively; for the public under 30, more efforts should be made to enable them to understand the issue of “Animal Welfare and Rights”. Non-governmental organizations, wildlife conservation organizations, and enterprises should also be encouraged to hold various thematic education activities [54], such as watching public welfare videos, so that the audience can have a deeper understanding of the meaning of the theory of the SWM and the role it plays in the field of wildlife conservation across the world in a more direct way. In addition, as social media tends to promote eye-catching but less scientific information, but little about sustainable wildlife conservation and management [54], relevant experts should be encouraged to speak on wildlife conservation issues on social media.

## 5. Conclusions

The results of this study can be used to help Chinese wildlife managers develop more scientific policies and practices for wildlife conservation and management and highlight the critical role that education must play if science-led conservation policy and practice is to survive as the dominant paradigm. In the future, it is imperative that conservation managers and policy makers are better informed about societal attitudes so that future decisions better reflect public sensitives and avoid the potential for avoidable conflict.

This is the first research concerning the attitudes of the urban public in China to SWM and wildlife conservation. Due to the nature of the survey and the restrictions imposed by COVID-19, this study was not able to explore views and attitudes more deeply, and in the future, we recommend that the questionnaire survey is supplemented by semi-structured interviews to probe more deeply and to gain an understanding of what may or may not be considered to be acceptable management practice and why. In future studies, the form of the survey questions in the questionnaire could be updated to understand the public’s attitude more clearly and intuitively, for example, including some questions with obvious “model/textbook” answers as “controls” of sorts, and also incorporate in-depth interviews with a subsample to help contextualise and elaborate on the views that emerge from the questionnaire. It would also be informative to compare and contrast urban views with rural households to identify where the greatest conflict and synergies exist for conservation management. By so doing, a richer, more nuanced, and more representative picture of public attitudes to wildlife management in China will emerge.

## Figures and Tables

**Table 1:** Questions used to measure respondents’ attitudes toward sustainable wildlife management and conservation.

Questions of High Relevance to SWM Composing Each Issue
***Animal Release* ^c^**
1. People should be able to release wild animals at will. ^b^
13. Wildlife should be released by professional departments or organizations. ^a^
15. Buying wildlife from the market and releasing them is beneficial to conservation. ^b^
***Animal Welfare and Rights***
2. Animals should have equal rights with humans. ^b^
10. Advocating animal rights is more important than utilization of wildlife by men. ^b^
16. Animal welfare should be improved in captive breeding of wildlife. ^a^
***Wildlife Utilization and Captive Breeding***
3. As long as the use of wildlife and their products is prohibited, wild animals can be effectively conserved. ^b^
18. All wild animals and their products, including captive breeding of wild animals and their products, should not be used. ^b^
***People and Wildlife Management***
4. If wild populations are not threatened, we can use wildlife and their products to improve people’s quality of life. ^a^
8. The standard of living of residents around the wildlife distribution area should be considered in wildlife conservation. ^a^
20. Wildlife management should be based on science. ^a^
***Vegetarianism and Wildlife Conservation***
5. Vegetarianism is not an effective way to conserve and manage wildlife. ^a^
11. Vegetarianism is closely related to wildlife conservation. ^b^
17. Wild animals can be effectively conserved if all the human beings are vegetarians. ^b^
***Public Attitudes to Wildlife Conservation***
6. If all people have a positive (scientific and rational) attitude about wildlife, wildlife will be effectively conserved. ^a^
14. Wildlife can be conserved as long as they are given care and love. ^b^
19. No matter how enthusiastic we care about wildlife, we still need scientific methods for its conservation. ^a^
***Trophy Hunting***
7. Trophy hunting is cruel and inhumane to animals. ^b^
9. Well-managed trophy hunting is one of the effective measures for wildlife conservation and management. ^a^
12. Well-managed trophy hunting opportunities should be provided for those who want to hunt. ^a^

^a^ The scoring system of these questions was: −3 “strongly disagree”; −2 “disagree”; −1 “slightly disagree”; 0 “not bothered/do not know”; +1 “slightly agree”; +2 “agree”; +3 “strongly agree”. ^b^ The scoring system of these questions was: +3 “strongly disagree”; +2 “disagree”; +1 “slightly disagree”; 0 “not bothered/do not know”; −1 “slightly agree”; −2 “agree”; −3 “strongly agree”. ^c^ Release refers to the human activity of reintroducing endangered or injured species into the natural environment.

**Table 2:** Multivariate stepwise linear regression analysis of public attitudes about sustainable wildlife management and conservation.

	Unstandardized Coefficients		Standardized Coefficients	t	Sig.	Rank	Collinearity Statistics	
Model	B	Std. Error	Beta				Tolerance	VIF
(Constant)	0.716	0.054		13.199	0.000			
Vegetarianism	−0.296	0.031	−0.195	−9.616	0.000 **	1	0.919	1.088
Education	0.094	0.011	0.170	8.546	0.000 **	2	0.961	1.041
Religion	−0.215	0.033	−0.131	−6.553	0.000 **	3	0.950	1.053
Age	−0.052	0.009	−0.112	−5.702	0.000 **	4	0.984	1.016
Gender	−0.081	0.022	−0.071	−3.636	0.000 **	5	0.994	1.016
Level of City	−0.036	0.010	−0.070	−3.566	0.000 **	6	0.984	1.016
Salary	0.016	0.009	0.037	1.647	0.100	7	0.753	1.327
F				54.936				
P				0.000				
Dependent Variable: Overall attitude about sustainable wildlife management and conservation								

Statistically significant differences (p < 0.01) are marked with **.

## Data Availability

The data presented in this study are available on request from the corresponding author. The data are not publicly available due to privacy and ethical restrictions.

## Supplementary Materials

The following are available online at https://www.mdpi.com/article/10.3390/ani11092521/s1, Table S1: Percentage of respondents in each research area, Table S2: Summary of respondent demographics. Table S3: Overall Attitudes of public toward sustainable wildlife management and attitudes toward seven categories of issues and twenty questions.
