# Supplementary material for: Compassionate Conservation and the Challenge of Sustainable Wildlife Management: A Survey of the Urban Public of China
Source: Animals (Basel). 2021 Aug 27;11(9):2521. doi: 10.3390/ani11092521 (PMC8468314; doi:10.3390/ani11092521)
Supplement: Supplementary file 1 [file animals-11-02521-s001.zip › animals-1313545-supplementary.pdf]

animals

Supplementary Materials: Compassionate Conservation and the challenge of Sustainable Wildlife Management: A Survey of the Urban Public of China

Zhen Miao [1,†], Qiang Wang [2,†], Xinyi Lu [1], Dongxiao Chen [1], Wei Zhang [1,*], Xuehong Zhou [1,*] and Douglas C. MacMillan [3]

[1] College of Wildlife and Protected Area, Northeast Forestry University, Harbin 150040, China; miaozhen43566@163.com (Z.M.); luxinyi981125@163.com (X.L.); dongxiao_c@163.com (D.C.)
[2] Key Laboratory of Wetland Ecology and Environment, Northeast Institute of Geography and Agroecology, Chinese Academy of Sciences, Changchun 130102, China; qwang@neigae.ac.cn
[3] Durrell Institute of Conservation and Ecology (DICE), University of Kent, Canterbury, Kent CT2 7NR, UK; dcm@kent.ac.uk
* Correspondence: zwfur@nefu.edu.cn (W.Z.); xuehong_zhou2012@nefu.edu.cn (X.Z.)
† Equal contributions.

Received: 9 July 2021; Accepted: 23 August 2021; Published: 27 August 2021

Table S1. Percentage of respondents in each research area.

Seven regions	City	Number of questionnaires collected	Number of questionnaires completed
North China	Beijing	262	200
	Datong	139	100
Eastern China	Hangzhou	183	178
	Yantai	128	122
	Zhucheng	120	120
South China	Guangzhou	194	94
		258 (online)	206
Central China	Wuhan	115	95
	Zhengzhou	255 (online)	205
Northeast China	Harbin	213	196
	Genhe	150	148
Northwest China	Xining	149	133
	Xi'an	126 (online)	103
	Xinjiang Uygur Autonomous Region He Jing County	147	110
Southwest China	Chongqing	121	80
	Chengdu	293 (online)	220
Total		2853	2310

Table S2. Summary of respondent demographics.

Demographic	Variable	N	%
Living area	North China	300	13
	Eastern China	420	18.2
	South China	300	13
	Central China	300	13
	Northeast China	344	14.8
	Northwest China	346	15
	Southwest China	300	13
Gender	Male	1205	52.2
	Female	1105	47.8
Age	≤20	381	16.5
	21-30	590	25.5
	31-40	587	25.4
	41-50	571	24.7
	51-60	139	6
	≥61	42	1.8
Education	Junior high school and below	189	8.2
	High school (Secondary school)	673	29.1
	College	712	30.8
	Undergraduate degrees	622	26.9
	Postgraduate degree	114	4.9
Monthly salary	≤4000	962	41.6
	4000-6000	674	29.2
	6000-8000	298	12.9
	8000-10000	182	7.9
	10000-15000	115	5
	≥15000	79	3.4
Are you vegetarian?	Yes	397	17.2
	No	1913	82.8
Do you have religious beliefs?	Yes	327	14.2
	No	1983	85.8
The development level of the cities	First-tier cities	500	21.6
	New first-tier cities	881	38.1
	Second-tier cities	318	13.8
	Third-tier cities and below	611	26.5

Table S3. Overall Attitudes of public toward sustainable wildlife management and attitudes toward seven categories of issues and twenty questions.

	Item	Mean (Min, Max)	
Release	Q1: people should be able to release wild animals at will	1.105 (-3, 3)	1.147 (-2.67, 3)
	Q13:wildlife should be released by professional departments or organizations	1.856 (-3, 3)	
	Q15:buying wildlife from the market and releasing them is beneficial to conservation	0.481 (-3, 3)	
Animal Welfare and Rights	Q2:animals should have equal rights with humans	-0.844 (-3, 3)	-0.199 (-3, 3)
	Q10:advocating animal rights is more important than utilization of wildlife by men	-0.504 (-3, 3)	
	Q16:animal welfare should be improved in captive breeding of wildlife	0.750 (-3, 3)	
Utilization and Wildlife Conservation	Q3:as long as the use of wild animals and their products is prohibited, wild animals can be effectively conserved	-0.415 (-3, 3)	-0.255 (-3, 3)
	Q18:all wild animals and their products, including captive breeding of wild animals and their products, should not be used	-0.095 (-3, 3)	
Wildlife Management	Q4:if wild populations are not threatened, we can use wildlife and their products to improve people's quality of life	-0.663 (-3, 3)	0.828 (-2.67, 3)
	Q8:the standard of living of residents around the wildlife distribution area should be considered in wildlife conservation	1.096 (-3, 3)	
	Q20:wildlife management should be based on science	2.051 (-3, 3)	
Vegetarianism and Wildlife Conservation	Q5:vegetarianism is not an effective way to conserve and manage wildlife	1.225 (-3, 3)	0.763 (-3, 3)
	Q11:vegetarianism is closely related to wildlife conservation	0.230 (-3, 3)	
	Q17:wild animals can be effectively conserved if all the human beings are vegans	0.836 (-3, 3)	
Public and Wildlife Conservation	Q6:if all men have a positive attitude about wildlife, wildlife can be effectively conserved	1.763 (-3, 3)	1.157 (-2, 3)
	Q14:wildlife can be conserved as long as given care and love	-0.440 (-3, 3)	
	Q19:no matter how enthusiastic we care about wildlife, we still need scientific methods for its conservation	2.148 (-3, 3)	
Trophy Hunting	Q7:trophy hunting is cruel and inhumane to animals	-1.348 (-3, 3)	-0.475 (-3.3)
	Q9:well-managed trophy hunting is one of the effective measures for wildlife conservation and management	-0.352 (-3, 3)	
	Q12:well-managed hunting opportunities should be provided for those who want to hunt	0.276 (-3, 3)	
Total average score of the public attitudes toward SWM and wildlife conservation: 0.458 (-1.3, 2.9)			
